# How have temporary Medicare telehealth item numbers impacted the use of dietetics services in primary care settings?

**DOI:** 10.1111/1747-0080.12743

**Published:** 2022-06-12

**Authors:** Jaimon T. Kelly, Alireza Ahmadvand, Centaine Snoswell, Lauren Ball

**Affiliations:** ^1^ Centre for Online Health, Faculty of Medicine The University of Queensland Brisbane Queensland Australia; ^2^ Centre for Health Services Research, Faculty of Medicine The University of Queensland Brisbane Queensland Australia; ^3^ School of Medicine and Dentistry, Griffith University, Gold Coast Campus Southport Queensland Australia; ^4^ Department of Gastroenterology & Hepatology Princess Alexandra Hospital Brisbane Queensland Australia; ^5^ Menzies Health Institute Queensland, Griffith University, Gold Coast Campus Southport Queensland Australia

**Keywords:** COVID‐19, diet, nutrition care, primary care, medicare, telehealth

## Abstract

**Aim:**

The aim of the study was to describe the quantity and cost of in‐person and telehealth dietetics services reimbursed under Australia's Medicare Benefits Scheme, before and during the coronavirus pandemic.

**Methods:**

Publicly available Medicare Benefits Scheme dietetics service activity data were extracted from an online database, between January 2019 and June 2021. For allied health telehealth items, it was assumed that between 10% and 20% of all consults were dietetic related.

**Results:**

Dietetics service claims reimbursed through the Medicare Benefits Scheme averaged 115 thousand per quarter in 2019. In quarter 2 of 2020, service delivery dropped by 25% compared to quarter 1 of 2020 and 32% compared to 2019. This drop recovered in quarters 3 and 4, with dietetic consultations claimed through the Medicare Benefits Scheme remaining relatively comparable to 2019 data. Dietetics services cost AUD 5,868,021 in quarter 1 2019 and AUD 5,742,632 in quarter 1 2020. Since the introduction of allied health telehealth items, the number of consultations claimed per quarter has accounted for between 17.7% (quarter 2 2020) and 4.5% (quarter 2 2021) of all consultations per quarter.

**Conclusions:**

The provision and costs of dietetics services in Australia have remained relatively constant compared to 2019 data, indicating telehealth was being used for substitutive rather than additive care, apart from an initial reduction of 25% between March and June 2020. The introduction of telehealth items for dietitians has been modest, peaking at 17.7% and now consistently averaging 5% of total dietetics services. The permanent implementation of telehealth items is unlikely to cause significant increases in cost or access and will assist Australians to eat better to support improved chronic disease outcomes.

## INTRODUCTION

Poor diet is recognised as the most common modifiable risk factor for chronic disease, causing an estimated 350 000 years of healthy life lost in 2015.^1^ Over 93% of Australian adults do not eat the recommended daily serves of vegetables which significantly increases their risk of developing chronic disease.^1^ Dietitian services are fundamental for preventing and managing chronic diseases in the community. In the past 15 years, the number of dietitians operating in primary care has more than tripled, signifying increased demand from community members for support to eat well.^2^

One of the most significant investments by the Australian Government in chronic disease management is the Enhanced Primary Care (EPC) program introduced in 1999, which expanded to the Chronic Disease Management (CDM) program in 2004.^3^ Under this program, a general practitioner (GP) can refer an individual to a range of allied health practitioners for up to five subsidised consultations per calendar year,^3^ including dietitians. In an evaluation of private practice dietetics services between 2004 and 2013, dietitians were the third most commonly referred to allied health professional in the CDM program.^4^

With the 2020–21 public health efforts to mitigate the impact of the novel coronavirus (COVID‐19) pandemic, this made access to face‐to‐face appointments more challenging, regardless of geographic location. Travel restrictions, public health orders to maintain social distancing, self‐isolation requirements, and advice to avoid non‐essential medical activities substantially impacted health utilisation all over the world, resulting in an estimated 37% reduction in total healthcare utilisation between February and May 2020 across more than 10 countries worldwide.^5^ Furthermore, people have been more likely to avoid healthcare settings if they had minor illnesses or did not perceive a service as lifesaving, and it remains to be seen what long‐term impact this may have on individual and population health.^5^ In an attempt to mitigate this risk, the Australian Government announced temporary financial support through the Medicare Benefits Schedule (MBS) to allow people who would otherwise be eligible to use the CDM program to be able to access services through phone and videoconference modalities.^6^ This significant change in policy was coupled with the introduction of telehealth item numbers on the MBS for dietitians to deliver eating disorder consultations, first introduced in November 2019.^7^ Whilst this support has been a welcomed policy direction, it remains unclear how dietetics service utilisation has been impacted, whether telehealth MBS items are sustainable as a permanent component of Medicare.

The aim of this study was to describe the quantity and cost of in‐person and telehealth dietetics services reimbursed under the MBS, before and during the coronavirus pandemic (2019–June 2021).

## METHODS

This was an ecological study involving population‐level MBS data to describe dietetics services reimbursed by Medicare between January 2019 and June 2021,^8^ reported using descriptive statistics. Ethical exemption was granted by The University of Queensland's Human Research Ethics Committee (2021/HE002244).

All MBS publicly available data for dietetics services delivered in‐person, by videoconference or phone, were accessed from the Medicare Australia website, provided by the Australian Government. The database is an accurate and reliable representation of all publicly‐funded services in Australia. Services examined included general dietetic consultations performed by an Accredited Practising Dietitian (APD) and referred from a GP, for individual, group assessment and follow up, specific Indigenous, eating disorders, and residential aged care consultations.^9^ Telehealth consultations, defined by the MBS as telephone and videoconference consultations performed by a dietitian to a patient, were also examined. A full list of the extracted codes and their associated introduction time is presented in Table S1 (supplementary material).

Data were exported from the Medicare Australia website to Microsoft Excel (2018, Microsoft Corp.) for handling and cleaning, prior to analysis. Rates of service provision were reported as quantity of, and cost for, services for each quarter of the year. Descriptive analyses were conducted and involved calculating quarterly totals, means for monthly totals, and proportion of videoconference, telephone and telehealth modalities as percentages. Monthly services were graphed by delivery mode (in‐person, videoconference and phone).

Some of the temporary telehealth item numbers for allied health consultations (item numbers 93000, 93013, 93048 and 93061) did not delineate between different allied health providers’ speciality (see ‘*’ in Table S1 for each of these item numbers). Therefore, to approximate the quantity and cost of allied health telehealth services which were conducted by dietitians, a series of dietetic telehealth scenarios, using descriptive analysis and an assumption that dietitian services would account for 10%–20% of all allied health MBS data, were used. This assumption was conservatively made based on data showing that dietitian consults make up 7% of all in‐person appointments for allied health EPC referrals^4^ and all other allied health (except for podiatry and physiotherapy) make up a collective total of ~25% of total allied health EPC referrals^10^ (codes with * in Table S1 signify where these assumptions are used). Three scenarios were then modelled where the total allied health phone and video consultations (item numbers 93000, 93013, 93048 and 93061) would be conducted by dietitians and compared this to the change in in‐person dietetic MBS claims (item numbers 10954 and 81320) to define a proportion of this change which would have been driven by telehealth uptake during the observational time period.

Data on MBS claims and costs were exported to Microsoft Excel and were analysed using simple descriptive statistics (counts and percentages). All data analyses were conducted in Microsoft Excel.

## RESULTS

Prior to the COVID‐19 pandemic, dietetics service claims through the MBS averaged 115 thousand per quarter (Q) in 2019 (Table 1). At the onset of the pandemic, videoconference and phone items became available mid‐way through March 2020. Despite this, there was a reduction in services in Q2 2020. In 2020, MBS claims were less consistent per quarter. Specifically, in Q1, total claims averaged 35363 consultations per month (an 8% decrease from Q4 of 2019). The largest fall in service delivery occurred in Q2 of 2020 (April–June; at the height of the first wave of the pandemic in Australia), where the average number of dietetics services dropped to 26638 consultations per month, representing a decrease of 25% from Q1 of 2020 and 32% compared to Q2 of 2019. This rate recovered in Q3, with dietetic consultations claimed through MBS representing only a 1% reduction compared to Q3 of 2019. Total dietetics services increased in Q4 of 2020 by 8% (and a 10% increase compared to Q4 of 2019), which was sustained into the first 2 quarters of 2021 (Table 1).

**TABLE 1:** Quarterly data for dietetics services (MBS items) reimbursed by Medicare between 2019 and June 2021

	2019				2020				2021	
	Q1	Q2	Q3	Q4	Q1	Q2	Q3	Q4	Q1	Q2
*Number of consultations*										
Quarter total in‐person, *n*	110 785	117 456	117 347	114 668	105 959	65 801	105 430	116 711	118 849	121 969
Quarter total videoconference^a^, *n*	NA	NA	NA	NA	37 [28;46]	6335 [5182;7489]	5871 [5109;6633]	5387 [4876;5897]	3415 [3107;3723]	3465 [3210;3720]
Quarter total phone^a^, *n*	NA	NA	NA	NA	91 [66;117]	7779 [5418;10 140]	5287 [3736;6838]	4239 [2980;5497]	2705 [1940;3471]	2280 [1653;2906]
Quarter total, *n*	110 785	117 519	117 410	114 731	106 088	79 915	116 588	125 836	124 969	127 714
*Proportion of consultations by modality*										
Videoconference, %	NA	NA	NA	NA	0.0%	7.9%	5.0%	3.9%	2.7%	2.7%
Phone, %	NA	NA	NA	NA	0.1%	9.7%	4.5%	3.4%	2.2%	1.8%
Telehealth, %	NA	NA	NA	NA	0.1%	17.7%	9.6%	7.3%	4.9%	4.5%
Average monthly total, *n*	36 928	39 173	39 137	38 244	35 363	26 638	38 863	41 945	41 656	42 571

Abbreviations: Q1, quarter 1; Q2, quarter 2; Q3, quarter 3; Q4, quarter 4; *n*, number; MBS, Medicare Benefits Schedule.

^a^ Assuming 15% of Allied Health general consultation codes were performed by dietitians (item numbers 93000, 93013, 93048 and 93061), range of 10%–20% presented in [].

**TABLE 2:** Quarterly data for each itemised MBS telehealth dietetics services between 2019 and June 2021

Dietetics service delivered	Delivery mode	Item no.	2020				2021	
			Q1	Q2	Q3	Q4	Q1	Q2
Assessment for group services	In‐person, *n* (% of total)	81120	356 (100%)	295 (95.2%)	278 (83.5%)	272 (89.8%)	404 (87.1%)	165 (86.4%)
	Videoconference, *n* (% of total)	93284	NA	1 (0.3%)	16 (4.8%)	4 (1.3%)	3 (0.6%)	5 (2.6%)
	Phone, *n* (% of total)	93286	NA	14 (4.5%)	39 (11.7%)	27 (8.9%)	57 (12.3%)	21 (11.0%)
Group service	In‐person, *n* (% of total)	81125	476 (100.0%)	164 (89.6%)	521 (94.4%)	607 (98.9%)	394 (95.9%)	547 (100.0%)
	Videoconference, *n* (% of total)	93285	0 (0.0%)	19 (10.4%)	31 (5.6%)	7 (1.1%)	17 (4.1%)	0 (0.0%)
Eating disorder service ≥20 min (introduced in November 2019)	In‐person, *n* (% of total)	82350	4054 (85.4%)	3891 (52.9%)	6300 (61.3%)	6797 (62.0%)	7198 (71.6%)	8381 (71.4%)
	Videoconference, *n* (% of total)	93074	10 (0.2%)	2873 (39.0%)	3550 (34.5%)	3821 (34.8%)	2481 (24.7%)	2677 (22.8%)
	Phone, *n* (% of total)	93108	681 (14.4%)	595 (8.1%)	435 (4.2%)	352 (3.2%)	375 (3.7%)	681 (5.8%)
Individual care recipient in a residential aged care facility^a^	In‐person	93528	NA	NA	NA	0	0	0
	Videoconference	93537	NA	NA	NA	0	0	0
	Phone	93538	NA	NA	NA	0	0	3
Individual care recipient in a residential aged care facility of Aboriginal or Torres Strait Islander descent^b^ Introduced in November 2020	In‐person	93583	NA	NA	NA	0	0	0
	Videoconference	93592	NA	NA	NA	0	0	1
	Phone	93593	NA	NA	NA	0	0	0

Abbreviation: NA, not applicable, quarter pre‐dates code introduction; MBS, Medicare Benefits Schedule

^a^ Introduced in December 2020.

^b^ Introduced in November 2020.

Some of the increases in MBS activity observed in the first 2 quarters of 2021 were due to the introduction of new eating disorder MBS items in the fourth quarter of 2019. These new services gradually increased from 4745 consultations in Q1 2020 to 10054 in Q2 2021 (Table 2). Other new MBS items were introduced in December 2020 for services provided into residential aged care facilities; however, there have only been 5 claims since their introduction. Comparing MBS dietetic consultations made in Q1 and Q2 of 2021 to 2019 without the eating disorder items showed a 4% increase and 1% decrease, respectively, suggesting there has been no meaningful change in dietetics services uptake as a result of telehealth items for CDM and group‐based dietetics services.

**FIGURE 1:**
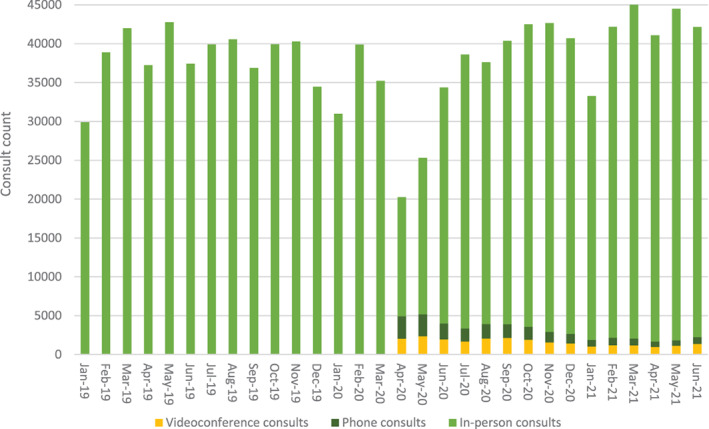
Monthly claimed Medicare dietetics services from January 2019 to June 2021, broken down into in‐person, phone and videoconference consultations (assuming 15% of allied health codes were dietetics services)

The uptake of telehealth item numbers is summarised in Table 2. Since the introduction of allied health telehealth items in March 2020, the number of consultations claimed per quarter has accounted for between 17.7% (Q2 2020) and 4.5% (Q2 2021) of all consultations per quarter. The greatest uptake of telehealth (17.7% of all consults for both phone and videoconference consultations) also corresponded to this quarter (Figure 1). Figure 1 provides a graphical presentation demonstrating the overall trend for both activities across Australia from January 2019 to June 2021. The first noticeable decline in service provision rates occurred between April and May 2020, corresponding to the initial height of the COVID‐19 pandemic.

Assuming a total proportion of 15% (10%, 20% reported in parenthesis) of total allied health telehealth conducted by dietitians, there was a peak of 18% in Q1 2020, dropping to a level of 5% in Q1 through Q2 of 2021. The adoption of phone compared to videoconference consultations has been relatively similar. Initially, phone consultations dominated videoconference in Q1 2020 after the items started on 13 March and reduced to 55 percent of all telehealth consultations in the following quarter. However, videoconference consultations have made up greater than 50 percent of telehealth consultations from that point forward to date (Table 2).

As the total estimated costs only relate to MBS reimbursement, the trend and changes in costs mirror the claims data reported above (Figure 1). Dietetics services cost Medicare AUD 5,868,021 in Q1 2019, AUD 5,742,632 in Q1 2020 and AUD 6,880,841 in 2021 (assuming 15% of allied health consultations were dietetics) (Figure 1). During Q2 in 2020 when the use of telehealth reached its peak, it accounted for 19% of the cost of dietetics services provided by Medicare. Phone consultations accounted for approximately AUD 425,000 of the AUD 4.4 million total cost in this quarter, whilst videoconference consultations accounted for approximately AUD 379,000. This reduced to AUD 241,000 for telephone consultations (2% of the total quarterly cost) in Q2 2021 and AUD 127,000 for videoconference consultations (3%).

## DISCUSSION

**FIGURE 2:**
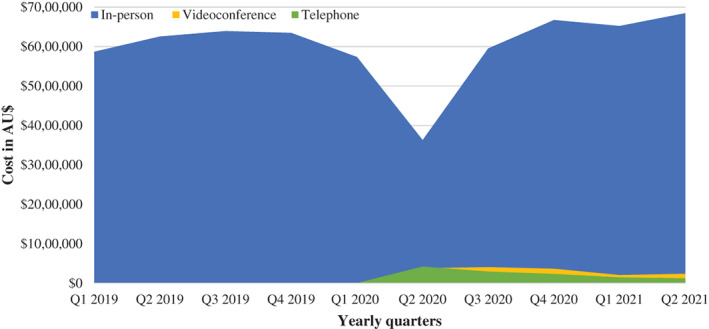
Monthly cost of Medicare reimbursement for dietetics services between January 2019 and June 2021, delineated by in‐person, phone and videoconference consultations (assuming 15% of allied health codes were dietetics services)

This study aimed to describe the quantity and cost of in‐person and telehealth (videoconference and phone) dietetics services reimbursed by Medicare, before and during the (current) COVID‐19 pandemic in Australia. The primary findings are that, apart from an initial reduction in dietetics services in March–April 2020 coinciding with the onset of the pandemic, the provision of dietetics services in Australia and their associated cost has remained relatively constant (Figures 1 and 2). The early reduction was observed across all Medicare services, with a reported reduction in non‐hospital services from approximately 34 million in March 2020 to 29 million in April 2020.^11^ Telehealth consultations offered by videoconference and phone have become part of routine practice since the temporary codes were announced in March 2020. In fact, it was recently announced by the Australian Department of Health that all allied health (including dietetics) telehealth‐delivered primary care services will remain permanent.^12^ Additionally, the constant nature of the overall number and cost of claims indicates that telehealth is primarily being used for substitutive rather than additive care. This potentially dispels speculation that allied health telehealth services would result in unfeasibly large cost increases.

The findings show that the introduction of telehealth MBS item numbers has allowed dietitians in primary care to continue to function as usual and deliver continuous dietetic care to all Australians. The pattern of eating disorder item numbers (which sit outside the CDM and group‐based consultations and were first introduced in November 2019^7^) is still steadily increasing since their introduction, at the time of writing. Thus, including these claims, the total estimated dietetics services are up ~10% on 2019 MBS claims, but when removed, the difference in total dietetic claims (and the costs associated with these claims) has remained comparable to 2019.

These findings contribute to the evidence base suggesting that continuing telehealth item numbers permanently for dietitians in Australia is beneficial for continuing health access in primary care. However, further investigation is needed to test the effects of this policy change on improving dietetic care access and outcomes in primary care. The evidence base for telehealth‐delivered dietetic care continues to grow, showing that these interventions are cost‐effective and demonstrate equivalent or improved outcomes as standard in‐person care.^6^, ^13^ Based on our current data, telehealth uptake has been apparently modest, suggesting it is being used as a substitution service rather than contributing to an increase to usual services. Therefore, the current study's data supports the rationale for continuing telehealth item numbers for dietitians by the Australian Government. This evidenced‐informed decision will continue to expand the opportunities for people to access dietetic care.

The modest uptake of telehealth shown, and substitutive nature it is being used for, may also be indicative of the fact that many vulnerable Australians are missing the opportunity to access essential dietetic care. This may create a ‘digital divide’ which could be exacerbated by the social determinants of health, which telehealth itself is not a cure‐all for. Therefore, this is an area of research, advocacy and promotion that requires much more work and attention.^14^

The COVID‐19 pandemic has challenged business continuity for health services, including primary care. Australia's primary care response to enable dietitians to temporarily deliver services via telehealth recognised that most vulnerable people require ongoing interactions and support to continue self‐managing their care during various public health restrictions. Primary care is Australia's panacea for chronic disease management, and for this reason alone, it could be argued that more is needed to improve health access via telehealth expansion and investment.^6^ Telehealth item numbers are part of the ‘function’ of the National Primary Care Targeted Action Plan, to preserve the functional capacity of the healthcare system.^15^ The *Medical Journal of Australia* together with VicHealth (a Public Health Promotion Foundation in Victoria, Australia) recently speculated on how Australia can become a healthy, fair and sustainable society by 2030, strongly advocating for investment in telehealth and digital health technologies to become more business‐as‐usual, as one of these key enablers.^16^

Telehealth is a supportive arm of primary care and is not a replacement for face‐to‐face services. The current study shows that the adoption of telehealth peaked in Q2 of 2020 at 18%, which has regressed to a relatively steady 5% throughout 2020 and 2021. This rate does contrast with other countries that have more experience with telehealth, however were also significantly challenged by the COVID‐19 pandemic. For example, in the US, approximately 11% of consumers used telehealth in 2019 compared to over 46% of consumers in 2020 who were using telehealth instead of in‐person visits to receive healthcare.^17^ However, we know that Australia has been slow to adopt telehealth. As an example, a previous analysis of pre‐COVID psychology‐related MBS claims (which have been implemented for longer than the temporary dietetics items) reveals that telehealth typically accounts for less than 2% of total MBS claims.^18^, ^19^ This likely indicates that the primary care ecosystem still has a way to go to be fully equipped for, and ready to embrace, telehealth delivery as part of routine care. Our results reveal that telehealth was not able to bridge the significant reduction in overall dietetics service utilisation during the onset of COVID‐19 and the associated public health mitigation efforts, with an estimated 25% reduction in total (including telehealth) dietetic care observed during the second quarter of 2020 compared to the same time in 2019. This is less than the 37% reduction in total health service utilisation reported in a recent systematic review of 81 studies reported across 10 countries.^5^ This review found the greatest reduction in healthcare utilisation between February and May 2020 to be all healthcare visits to a professional, which observed a median 42% reduction.^5^ These results show just how influential telehealth has been in Australian primary care during this time, without which the reduction in people accessing non‐hospital services may have been far greater.^11^

The Dietitians Australia position statement on telehealth calls for broader funding and eligibility for dietitians to provide the same high‐quality care they deliver in clinic rooms, remotely via telehealth.^6^ It has been shown that dietetic programs delivered via telehealth are a responsive and cost‐effective alternative or complement to traditional in‐person delivery of dietetics services, leading to comparative outcomes as observed in face‐to‐face care, when delivered in clinics, the community or in patients’ homes.^6^ Telehealth and digital health more broadly allow dietitians to deliver high‐quality medical nutrition therapy in novel and efficient ways which improve patient care.^20^ It is known that Australians are more embracing than ever of telehealth^21^ and a survey of registered dietitians during 2020 revealed them to likewise be highly accepting and embracing alternatives to in‐person clinic and even inpatient visits.^22^ The provision of specialist allied health services like dietetics using telehealth has other positive impacts for patients and clinicians beyond clinical benefits. For example, telehealth visits have given dietitians the opportunity for broader assessment, such as the ability to observe and assess a patient's home environment (such as refrigerators and pantries), allowing for a more comprehensive nutrition assessment.^22^ Telehealth has many extra‐clinical benefits like reducing travel for patients and clinicians, reducing the time away from usual activities for patients which minimises societal productivity losses, and increasing the accessibility of services for patients.^23^, ^24^

Our study has important limitations to consider. This study used publicly available MBS dietetic activity and costs data. These data, therefore, cannot determine the clinical effectiveness of publicly funded telehealth‐delivered dietetics services which could not be explored and should be a focus area for future research. Given the aggregate national nature of the data being used the generalisability to local areas and specific population groups is limited. The provision of in‐person dietetics services requires a local dietitian and therefore these services are more likely to have occurred in metropolitan or high‐population areas with actively referring general practitioners. Similarly, telehealth uptake requires both clinician and patient willingness in order for a consult to be conducted. Whilst these modalities may increase the accessibility of services for rural and remote individuals, it is not possible to determine the location of those who received services from the available MBS data. To provide an estimate of dietetics services only, it was assumed that 15% (10%–20%) of the broad allied health item numbers were claimed by dietitians. Whilst varying this number from 10% to 20% did not have a large impact on the totals provided, the assumption should be acknowledged. The costs described in the study are only those borne by Medicare and do not include out‐of‐pocket costs borne by patients or gap payments covered by private health insurers. Finally, the data presented here only represent publicly funded dietetics services, since many dietetics services are privately funded, the estimates here do not represent all dietetics services offered in Australia during 2019–2021.

The provision of dietetics services in Australia and their associated cost has remained relatively constant, aside from the initial onset of the coronavirus pandemic. In March–June 2020, there was a 25% reduction in total dietetics services, which was paralleled by an 18% increase in telehealth‐delivered dietetics services. Despite the introduction of new MBS items for videoconference, phone, eating disorder and residential aged care facility dietetics services over this time, the uptake and cost of Medicare claim reimbursements were similar across all quarters except the second quarter of 2020 which coincided with the pandemic onset. The relatively unchanged pattern in MBS claims does, however, suggest that telehealth may not be reaching the people who likely need dietetic care the most, and therefore, future research, advocacy and promotion are needed to ensure that telehealth improves healthcare access and lives up to its promise. These reliable data should give governments and decision makers assurance that telehealth item numbers for dietetics services are a sustainable function of Medicare, and these item numbers could become a permanent fixture of the MBS to support service continuity and better health access.

## AUTHOR CONTRIBUTIONS

JTK, AA, CS and LB contributed to the conception and design of this paper. CS extracted the data. JTK interpreted the data and drafted the manuscript. All authors contributed to revisions of the manuscript and read and approved the final version.

## CONFLICT OF INTEREST

All the authors have no conflicts of interest to declare.

## Supporting information


**Table S1** Data parameters used to export data from Medicare Australia (1).

## Data Availability

Data pertaining to this study are freely available on the Australian Government Medicare website.
