# Supplementary material for: How have temporary Medicare telehealth item numbers impacted the use of dietetics services in primary care settings?
Source: Nutr Diet. 2022 Jun 12;79(4):481–8. doi: 10.1111/1747-0080.12743 (PMC9539970; doi:10.1111/1747-0080.12743)
Supplement: Supplementary file 1 — Table S1 Data parameters used to export data from Medicare Australia (1). [file NDI-79-481-s001.docx]

Supplementary FILE

**Table S1:** Data parameters used to export data from Medicare Australia (1).

| **Mode** | **Code** | **Title** | **Introduction date** |
| --- | --- | --- | --- |
| In-person | 10954 | Dietitian CDM In-person (10954) | Ongoing |
| In-person | 81120 | Dietitian group assessment in-person | Ongoing |
| In-person | 81125 | Dietitian group in-person (81125) | Ongoing |
| In-person | 81320 | Dietitian ATSI in-person (81320) | Ongoing |
| In-person | 82350 | Dietitian eating disorder ≥20min in-person (82350) | 1/11/2019 |
| In-person | 93505 | Dietitian GP MCP RACF initial in-person (93505) | 10/12/2020 |
| In-person | 93528 | Dietitian GP MCP RACF follow up in-person (93528) | 10/12/2020 |
| In-person | 93550 | Dietitian ATSI & RACF initial in-person (93550) | 10/12/2020 |
| In-person | 93583 | Dietitian ATSI & RACF follow up in-person (93583) | 10/12/2020 |
| Videoconference | 93000* | AH CDM Telehealth COVID (93000) | 13/03/2020 |
| Videoconference | 93048* | AH ATSI VC (81320) | 30/03/2020 |
| Videoconference | 93284 | Dietitian group assessment VC | 22/05/2020 |
| Videoconference | 93285 | Dietitian group VC | 22/05/2020 |
| Videoconference | 93074 | Dietitian eating disorder ≥20min VC (93074) | 30/03/2020 |
| Videoconference | 93537 | Dietitian GP MCP RACF follow up VC (93537) | 10/12/2020 |
| Videoconference | 93592 | Dietitian ATSI & RACF follow up VC (93592) | 10/12/2020 |
| Phone | 93013* | AH CDM Phone COVID (93013) | 13/03/2020 |
| Phone | 93061* | AH ATSI phone (93061) | 30/03/2020 |
| Phone | 93108 | Dietitian eating disorder 20min+ phone (93108) | 30/03/2020 |
| Phone | 93286 | Dietitian group phone | 22/05/2020 |
| Phone | 93538 | Dietitian GP MCP RACF follow up phone (93538) | 10/12/2020 |
| Phone | 93593 | Dietitian ATSI & RACF follow up phone (93592) | 10/12/2020 |
| **Additional data export parameters** | | | |
| **Show** |  | Services as per capita |  |
| **Report format** |  | By time period (rows) and state (cols) |  |
| **Time period** |  | Months |  |
| **Start date** |  | January 2019 |  |
| **End date** |  | June 2021 |  |

Abbreviations: AH: Allied Health, ATSI: Aboriginal & Torres Strait Islander, CDM: Chronic disease management, COVID: Coronavirus, GP: General Practitioner, RACF: Residential aged care facility, MCP: Multidisciplinary care plan, VC: Videoconference.
*Assumptions are used for these codes, that dietitian services would make up for 10-20% of the total claims

**Reference**

1. Australia AG-S. Medicare statistics: Australian Government; 2021 [Available from: <https://www.servicesaustralia.gov.au/organisations/about-us/statistical-information-and-data/medicare-statistics>.
